# Supplementation with oxidised fish oil in pregnancy markedly increases neonatal mortality in male rat offspring

**DOI:** 10.1186/1687-9856-2015-S1-O41

**Published:** 2015-04-28

**Authors:** Benjamin Albert, Mark Vickers, Clint Gray, Clare Reynolds, José Derraik, David Cameron-Smith, Paul Hofman, Wayne Cutfield

**Affiliations:** 1 Liggins Institute, University of Auckland, New Zealand

## Introduction

Omega-3 fish oils are the most popular health supplements, taken by 15-30% of people at all stages of life, including pregnancy. These oils may improve insulin sensitivity through the recently characterized GPR120 receptor, but are chemically unstable and easily degrade into potentially toxic lipid peroxides. The health effects of oxidised fish oil are mostly unknown. We have shown that 89% of fish oil products in NZ exceed recommended oxidation limits, 36% by more than two-fold.

We hypothesised:

1. Fish oil supplementation during pregnancy of insulin-resistant mothers would prevent adverse metabolic programming of the offspring.

2. Oxidised fish oil would cause adverse metabolic and cardiovascular outcomes

## Methods

80 female Sprague-Dawley rats were allocated to a control or high-fat diet and time-mated. Throughout pregnancy they received 1ml of unoxidised fish oil, a highly oxidised fish oil, or control (water) by daily gavage. This dose of fish oil has been shown to reverse insulin resistance in rats. Neonatal mortality was recorded. Markers of oxidative stress and inflammation will be measured in unused pups. At weaning, male offspring were fed standard chow ad libitum. At 100 days they will undergo metabolic assessment including insulin sensitivity, lipid and inflammatory profiles, blood pressure and body composition.

## Results

Neonatal mortality was 8 times greater in the offspring of mothers treated with oxidised fish oil than controls (25.7 vs 3.2%, respectively; p<0.0001), with mortality from Day 2 to weaning being 1.4 times greater (p=0.042). Oxidised offspring had lower birth weight (5.4 vs 6.1 g p=0.008), and reduced weight at weaning (36.6 vs 40.7 g; p=0.07) and adolescence (day 51: 221.6 vs 256.7 g; p=0.02). Further, puberty was delayed compared with controls (day 45.3 vs 42.7; p<0.05).

## Discussion

Oxidised fish oil in pregnancy markedly increased neonatal mortality and had a powerful programming effect on the surviving offspring. Fish oils are often taken in pregnancy and are usually oxidised which may lead to adverse programming of growth and development. The long-term metabolic effects of the oxidised and fresh fish oil will be available for the ASM. As fish oils at retail are substantially oxidised, we recommend a precautionary response to these data: pregnant women should not take supplementary fish oil.

